# Erratum for Li et al., “Luciferase Immunosorbent Assay Based on Multiple E Antigens for the Detection of Chikungunya Virus-Specific IgG Antibodies”

**DOI:** 10.1128/spectrum.02282-22

**Published:** 2022-07-05

**Authors:** Xiaoxia Li, Xuan Wan, Jinyue Liu, Haiying Wang, Anan Li, Changwen Ke, Shixing Tang, Wei Zhao, Shaoxi Cai, Chengsong Wan

**Affiliations:** a BSL-3 Laboratory, Guangdong Provincial Key Laboratory of Tropical Disease Research, School of Public Health, Southern Medical University, Guangzhou, China; b Department of Respiratory and Critical Care Medicine, Nanfang Hospital, Southern Medical University, Guangzhou, Guangdong, China; c Department of Epidemiology, School of Public Health, Southern Medical University, Guangzhou, China; d Guangdong Center for Disease Control and Prevention, Guangzhou, China

## ERRATUM

Volume 10, no. 2, e01496-21, 2022, https://doi.org/10.1128/spectrum.01496-21. Page 1, author contribution line: “Xiaoxia Lia” should read “Xiaoxia Li.”

